# NO gas sensing kinetics at room temperature under UV light irradiation of In_2_O_3_ nanostructures

**DOI:** 10.1038/srep35066

**Published:** 2016-10-07

**Authors:** Nguyen Duc Chinh, Nguyen Duc Quang, Hyundong Lee, Truong Thi Hien, Nguyen Minh Hieu, Dahye Kim, Chunjoong Kim, Dojin Kim

**Affiliations:** 1 Department of Materials Science and Engineering, Chungnam National University, Daejeon 34134, Republic of Korea

## Abstract

In_2_O_3_ nanostructure sensors were fabricated by arc-discharging a source composed of a graphite tube containing indium. The NO gas sensing properties, as well as the morphology, structure, and electrical properties, were examined at room temperature under UV light illumination. In particular, the response and recovery kinetics of the sensor at room temperature under various UV light intensities were studied. The maximum response signal was observed at an intermediate UV light intensity, which could be corroborated by a nano-size effect based on the conduction model of a resistive chemical nano sensor. The mechanism for the enhanced adsorption/desorption kinetics for NO in an air environment under UV light irradiation is discussed in detail. Furthermore, the general requirements of the sensor, including the stability, repeatability, and selectivity, are discussed.

For the gas molecules to adsorb on the oxide surface, charge-exchange between the molecule and the surface atom is required. This ionosorption, or adsorption of molecules as ionic form via the charge-exchange, modifies the electrical conductance of the oxide material, thus leading to the sensor signal by the ratio of the material conductances before and after the gas exposure^1^,^2^,^3^. Since the ionosorption reactions are the surface phenomena, the nanostructural forms of the materials increase the sum of the surface reactions, and consequently, the sensor signals^4^,^5^,^6^,^7^,^8^,^9^. Indeed, high performance sensors were enabled by the nanostructural materials^10^,^11^,^12^. Besides of the material dimension, fast kinetics for adsorption and desorption processes are critical for good sensing properties. Since the physicochemical processes of the molecular adsorption and desorption on and from the surface are thermally activated processes, thermally activated surface processes enhance the sensor response and signal level. Most of the commercial semiconducting oxide gas sensors operate at high temperatures above 200 °C.

In this perspective, the resistive oxide sensor materials will show poor sensing properties, i.e. low signal level, slow response, and delay in recovery at room temperature (RT). However, if reasonable sensing performance can be obtained at RT, many advantages and benefits can be provided by the realization of advanced sensor systems^13^,^14^. For example, removing the power supplying circuits for heating is a huge advantage with regards to power consumption and integration into circuits. The low temperature operation allows reliable and safe sensing in explosive and flammable environments while ensuring the long-term stability of the sensors by negligible diffusion and sintering effects in the materials. The room temperature activity of chemical sensors is also highly demanded by the internet of things (IoT).

Photonic energy has been considered to replace the thermal energy required to promote surface reactions on the oxide surfaces without increasing the substrate temperature^15^. In many cases, nanoparticle- or nanowire-based structures have been employed, which will compensate for the low response levels of the sensing oxides at low temperatures. Several studies on photo-activated metal oxide gas sensors at RT have been reported. The common oxide sensor materials, such as ZnO^1^,^16^,^17^,^18^,^19^, SnO_2_^15^,^20^,^21^,^22^, TiO_2_^23^,^24^, WO_3_^25^, and In_2_O_3_^20^,^26^, have been extensively investigated for RT operation under the illumination of light. UV light irradiation results in an increase in the response signal, enhanced sensing reversibility, and an enhanced recovery rate. Such effects were observed for both oxidizing and reducing gases^13^,^17^,^18^,^19^,^20^,^23^. The wavelength and intensity of UV have been also examined in earlier reports^16^,^18^,^22^,^24^. While the effect of photon energy on the adsorption/desorption kinetics of gas molecules has been generally accepted, however, the underlying mechanisms, particularly, the correlation between photon energy and the structural properties, are not clearly understood yet.

Indium oxide (In_2_O_3_) is an n-type semiconductor that has a relatively high electrical conductivity in its non-stoichiometric form^27^. Single crystalline indium oxide has an energy gap of 3.5 eV, which can decrease to approximately 2.9 eV as its microstructure^28^,^29^. In this study, we developed a new synthesis method or co-arc-discharge method for indium oxide nanostructures. The method simultaneously employs the arc-discharge of carbon and indium using a graphite tube containing indium powder as the arc-discharge source. This co-arc-discharging of In and graphite produced running wires of agglomerated indium nanoparticles on the substrate mounted on the inside wall of the chamber. The as-fabricated porous In_2_O_3_ nanostructures were examined as a sensor for the detection of NO gas molecules at room temperature under UV light at 365 nm, which corresponds to ~3.4 eV. The UV light excites electrons from the valence band, which increases the electron and hole populations in In_2_O_3_ and supplies energy to the adsorbing and desorbing molecules on the surface, ultimately leading to fast adsorption and desorption kinetics. In this study, we scrutinized the light effect on the NO gas adsorption/desorption behaviors on and from In_2_O_3_ nanoparticles.

## Results and Discussion

### Device structure and morphology

**Figure 1:**
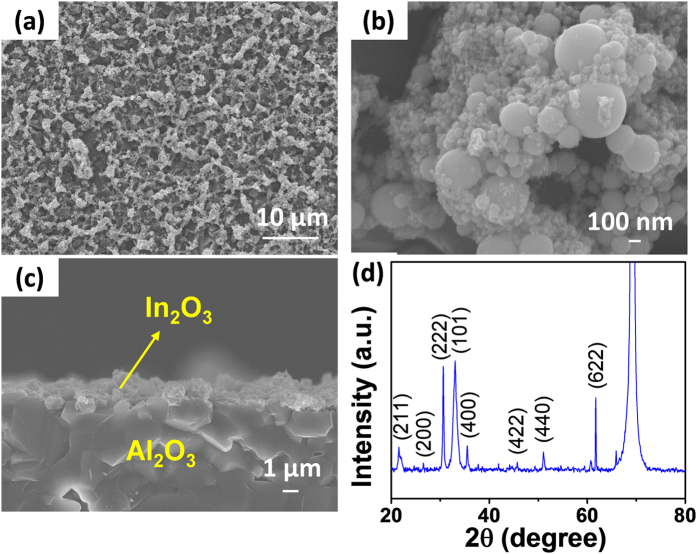
**(a–c)** Surface and cross-section SEM images of the In_2_O_3_ film. **(d)** X-ray diffraction spectrum of the In_2_O_3_ film.

Arc-discharge is a well-known method for the synthesis of both single- and multi-walled carbon nanotubes (CNTs)^30^,^31^,^32^. When a graphite rod containing catalytic metals is used as the arc-discharge source, the extremely high temperature involved in the arc-discharge process facilitates the synthesis of highly crystalline, single-walled CNTs via the vapor-liquid-solid synthesis route through the molten catalyst metal nanoparticles. Therefore, the catalyst metal particles are homogeneously distributed among the synthesized CNTs after processing. The catalyst metal particles that are distributed among the CNTs in the oxide form have been used to probe molecules in gases and liquids^33^,^34^. In this study, we further extended the arc-discharging method to the synthesis of non-catalytic metal nanoparticles. Arc-discharging of indium powder produced indium particles with various nanosizes, which were deposited on the inside wall of the arc-discharge chamber. The morphology is shown in Fig. 1a–c. Indium particles of various sizes that ranged from tens to hundreds of nanometers were produced and agglomerated to form micrometer scale hills running on the substrate. The indium vapors and/or spits formed via the arcing process may condense into nanoparticles of various sizes, which would lead to the observed morphology. Oxidation at 500 °C was expected to oxidize the indium particles and burn out the trace carbon in the structure. The XRD measurement confirmed the formation of crystalline In_2_O_3_ (Fig. 1d).

### Electrical properties

**Figure 2:**
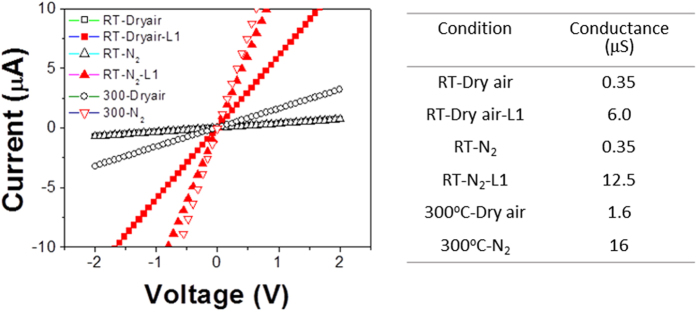
Current-voltage characteristics of the In_2_O_3_ nanostructure sensor measured under various conditions (i.e., gas environment, temperature, and UV illumination).

The current-voltage (I-V) characteristics of the fabricated In_2_O_3_ sensor structure were measured under various conditions, and the results are summarized in Fig. 2, where the effects of the temperature, environment, and UV light irradiation of L1 level were examined. Prior to the I–V measurements, the devices were heated to 350 °C in the given ambient conditions to desorb the preadsorbed oxygen and water molecules from the In_2_O_3_ surface. The measured I-V curves exhibited excellent linearity, indicating the formation of ohmic contact between the nanoparticles and the Au electrodes. The oxygen partial pressure in the gas environment as well as the substrate temperature determines the ionosorbed oxygen concentration on the surface, the carrier concentrations in the oxide, and the energy supplied to the surface reactions. The light also increases the carrier concentration in the oxide and supplies the activation energy for both the surface reactions of molecular adsorption/desorption and the chemical reactions.

The conductance of the structure measured at RT in dry air and N_2_ was ~0.35 μS with negligible differences. However, when the temperature was increased to 300 °C, the conductance increased to ~1.6 μS in dry air and ~16 μS in N_2_. Heating an n-type semiconducting material in air led to two competing processes as follows: the electron concentration increased due to thermal excitation and decreased due to the promoted oxygen ionosorption with electron capture on the surface. The comparison of the conductance between RT and 300 °C indicates that the thermal carrier generation effect is larger than the oxygen ionosorption effect at 300 °C. However, it should be noted that the amount of oxygen ionosorption at 300 °C is substantial. When the temperature was raised to 300 °C, thermally generated electrons in the oxide increased a conductance from 0.35 μS to 16 μS in N_2_ environment, however, only 1.6 μS could be achieved in the dry air environment due to the significant oxygen ionosorption on the surface that compensated 14.4 μS.

**Figure 3:**
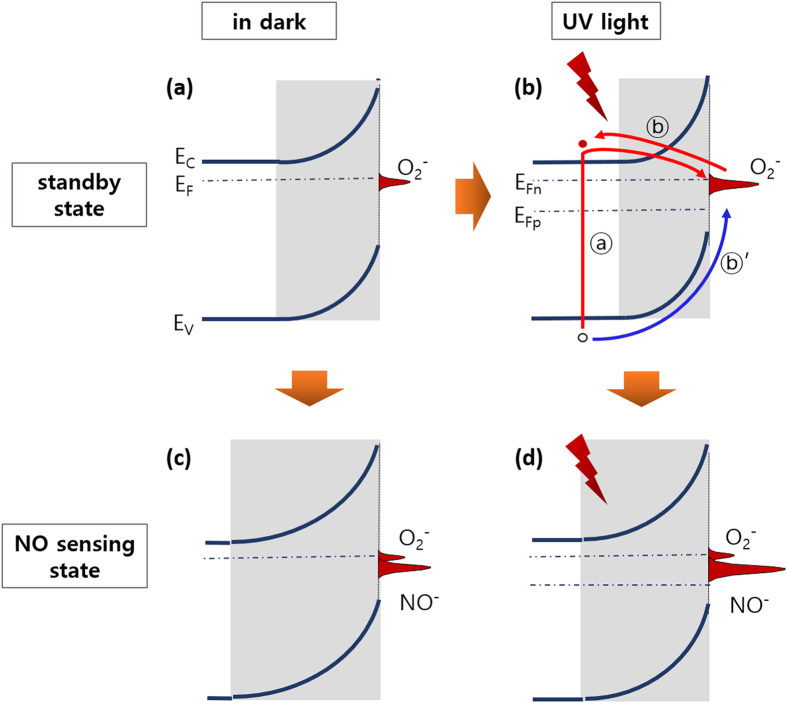
Schematic band diagram for the standby condition in air **(a)** in the dark and **(b)** under the UV light. The Fermi energy in the dark (E_F_) splits to the quasi Fermi energies for the electron and hole (E_Fn_ and E_Fp_) under the UV light. The carrier excitation processes due to light irradiation are shown by ① and ② as explained in the text. Both the electron and hole transitions are shown in the ② process. The band diagrams under NO exposure are shown **(c)** in the dark and **(d)** under the UV light. The relative variations of the adsorbed O_2_^−^ and NO^−^ charges are drawn. The variations in the depletion region depths in the dark and under the light before and after exposure to NO are also shown.

The effect of the UV light under different environmental conditions at RT was also studied. The conductance increased from 0.35 μS to ~12.5 μS in N_2_ and ~6 μS in dry air. Based on a similar logic, the conductance reduction under UV light was ~6.5 μS due to oxygen ionosorption, which could be estimated by the difference of the conductance between the different measurement environments (~12.5 μS vs. ~6 μS). Therefore, the UV light irradiation promoted not only electron generation but also oxygen ionosorption on the oxide surface at RT. Comparing the optical and thermal effects on the generation of carriers in N_2_ environment, the conductance increased to ~12.5 μS by the UV light irradiation and to ~16 μS by the heating to 300 °C, which indicates higher generation efficiency of the latter. On the contrary, the conductance in dry air under the light (~6 μS) is greater than at 300 °C (~1.6 μS). While the comparison reveals that the net carrier concentration in the light-irradiated material at RT is far higher than that the thermally generated ones at 300 °C, it confirms much higher oxygen ionosorption at 300 °C than RT. The change in the conductance and oxygen ionosorption via light irradiation can be expressed by the band diagrams shown in Fig. 3a,b, as discussed above. The results indicate two competing contributions to the conductance of the sensor structure from light irradiation (i.e., increased EHP (electron-hole pair) concentration and increased oxygen ionosorption).

### Temperature-dependent NO sensing properties of In_2_O_3_

**Figure 4:**
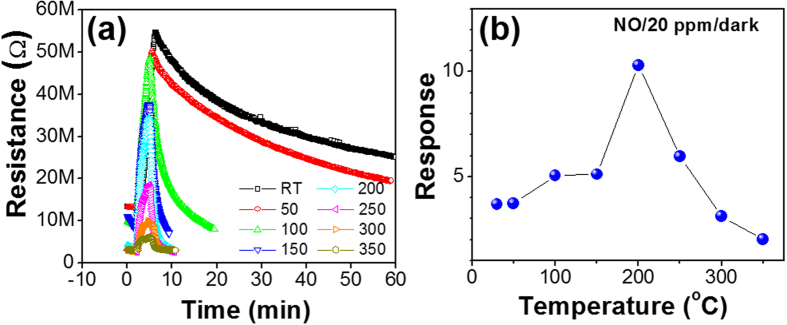
(a) Gas sensing properties of the In_2_O_3_ sensor upon exposure to 20 ppm NO at different working temperatures. **(b)** Gas response as a function of the working temperature.

The temperature-dependent NO gas sensing properties were measured at a NO gas concentration of 20 ppm at temperatures ranging from RT to 350 °C. The response-recovery curve measured at each temperature is plotted in Fig. 4a, and the response levels are plotted as a function of the substrate temperature in Fig. 4b. The increase in sensor resistance with exposure to the oxidizing gas (i.e., NO) confirms the n-type semiconducting behavior of In_2_O_3_. The maximum response of the sensor was 10.3, as observed at 200 °C. However, at RT, the maximum response of the sensor was 3.7. The large sensing signal or facilitated NO adsorption at 200 °C is the typical temperature dependence observed in many oxide semiconductor sensors, which is explained by the supply of the activation energy for the adsorption reaction and the facile electron supply to the adsorbing gas molecules^7^,^8^.

The finite and clear response at RT motivated us to further examine the sensing properties at RT due to the substantial advantages that are obtained from RT operation of the sensors. It is important to note that the critical limits of the RT performance are the slow adsorption kinetics, which can lead to non-saturating response signals, and the much slower desorption processes caused by the small thermal energy supplied at low temperatures. Therefore, UV light irradiation has been studied to replace the thermal energy for activation of the adsorption/desorption processes, thereby resulting in the improvement of the response and recovery at RT^13^,^18^,^20^.

### NO sensing properties of In_2_O_3_ at RT under UV light

**Figure 5:**
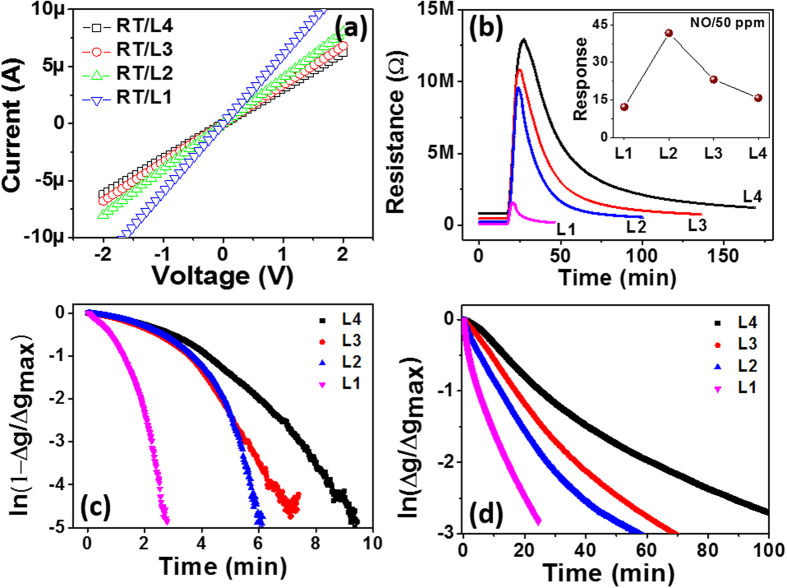
(a) Current-voltage characteristics of the In_2_O_3_ sensor measured in dry air under different UV irradiation conditions (L1, L2, L3, and L4). **(b)** Gas sensing properties measured at room temperature for 50 ppm NO under different UV irradiation conditions along with the signal levels. **(c,d)** Response and recovery times as a function of the UV light intensity.

UV irradiation intensity was varied from L1 to L4 during the conductance measurement (see experimental section for the detailed information). The RT conductance of the In_2_O_3_ sensor structure proportionally increased with the UV irradiation intensity, as shown in Fig. 5a. The current increase in proportion to the UV light intensity was caused by the proportionally increasing electron population in the conduction band. Next, the NO gas sensing properties of the In_2_O_3_ nanostructure were investigated upon exposure to 50 ppm NO gas diluted in dry air at RT under various UV light. A given UV light intensity was continuously irradiated on the sensor surface during the entire time period of the gas sensing measurement.

The baseline or standby conductance of the sensor was established by dynamic equilibrium between the adsorption and desorption of O_2_. With the introduction of NO flux into the test chamber, the NO molecules impinge on the oxide surface and are adsorbed until finally reaching the ultimate steady state of the response cycle. Such steady state is another dynamic equilibrium state established between the adsorbing fluxes of NO and O_2_ onto the surface and the desorbing fluxes of NO^−^ and O_2_^−^ out of the surface under irradiation^32^,^33^. Such adsorption reaction constant of NO is much larger (~10^5^) than that of O_2_ at high temperatures for many oxides such as WO_3_, ZnO, SnO_2_, etc.^8^,^18^,^35^, the high adsorption reactivity of NO can be achieved. The high response of In_2_O_3_ to NO is also attributed to its high reaction constant in the adsorption reaction.

The effects of UV light irradiation on the gas adsorption/desorption kinetics are described below. 1) The UV light excites electrons from the valence band (and donor levels) to the conduction band, leading to increase of electron and hole populations in the oxide (process ① in Fig. 3b). In this nonequilibrium state, the quasi Fermi energies (i.e., E_Fn_ and E_Fp_) can be defined by the electron and hole concentrations, respectively, in the band diagram of the oxide near the surface (Fig. 3b). The adsorption of NO (and O_2_) will be facilitated by the increased electron concentration because ionosorption of the oxidizing molecules requires capture of electrons from the oxide. The transitions are expressed in the band diagrams (change from Fig. 3a,b by oxygen adsorption in air and from Fig. 3c,d by NO adsorption in the sensing condition). This enhanced ionosorption due to irradiation results in a reduced activation energy for the adsorption reaction^7^,^8^. 2) The light can also excite electrons in the adsorbed NO^−^ (and O_2_^−^) (process ② in Fig. 3b), thus electron concentration in the sensor will increase. In parallel, the excess holes can facilitate the neutralization of NO^−^ 36 (process ② ’ in Fig. 3b). Both ② and ②’ processes neutralize NO^−^ (and O_2_^−^) to NO (and O_2_), thereby facilitate desorption of the molecules from the surface, especially during the recovery cycle, which results in a reduced activation energy for the desorption reaction. 3) The light can supply energy for electrons to overcome the energy barrier at the grain-grain interface and/or can modify the barrier height itself^24^,^37^,^38^. However, considering that the rate limiting step is the adsorption/desorption processes, this effect will be negligible in our sensor system.

Now, we will correlate the UV light effects with our measurements. Figure 5b shows the response-recovery curves measured for 50 ppm NO gas diluted in dry air at different illumination intensity levels (i.e., L1, L2, L3, and L4). By comparing the response/recovery curves measured with and without UV light (Figs 4a and 5b), i) irradiation with UV light greatly improved the response and recovery rates due to the fast adsorption and desorption rates for NO molecules, and ii) the maximum response level was observed at an intermediate UV light intensity (i.e., L2). The enhanced response kinetics can be explained by the facilitated supply of electrons to the adsorbing NO molecules due to process ①. The higher electron concentration in the oxide will lead to a higher probability for reaction with NO molecules, which will lead to the enhanced ionosorption of NO. Simultaneously, the empty sites provided by the enhanced desorption of preoccupied O_2_^−^ due to process ② can be predominantly occupied by NO due to its much higher adsorption reactivity compare to that of O_2_^8^. All of the processes accelerate the transition from Fig. 3b to d, leading to the higher response rate (i.e., adsorption rate) shown in Fig. 5c. Therefore, the higher UV light intensity led to a higher average response rate.

In the recovery process, the surface condition changes from Fig. 3d to b. The noticeably accelerated recovery rate under UV light irradiation resulted from the accelerated desorption of NO^−^ due to ② under halted NO impingement. Meanwhile, oxygen adsorption was promoted by process ①. Figure 5d indicates that the higher UV light intensity led to a higher average recovery rate (i.e., adsorption rate). Therefore, under UV light irradiation, the reactivity of the molecules at the surface was enhanced, leading to accelerated adsorption and desorption processes.

### Nanosize effect

Interestingly, the maximum sensor response was not observed with the highest UV light intensity (i.e., L1) but at the intermediate intensity of L2 (Fig. 5b inset). Similar results were observed in different oxide systems (i.e., ZnO and SnO_2_) but clear analyses were not performed^2^,^19^,^20^. In our study, this observation can be explained by the nanosize effect, similar to that discussed in detail in earlier reports^7^,^8^,^9^.

We reported that a resistive oxide nanowire sensor exhibits its maximum response when the diameter is near the total depletion condition or the critical depletion depth^7^,^8^ because the response is the *ratio* of the resistances before and after the adsorption of the analyte gas molecules. For the response given by *S* = *R*_*g*_*/R*_*o*_ (*R*_*g*_ and *R*_*o*_ are the resistance with and without the analyte gas, respectively) for an oxidizing gas, *R*_*g*_ sharply increases when the sensor approaches the total depletion condition, resulting in the highest response level, *S*. If the diameter is far greater than the critical depletion depth, the modulation of the conduction channel due to the oxidizing gas adsorption is small, therefore *S* is not much greater than 1. Whereas, if the diameter is smaller than the critical thickness, the sensor is already completely depleted prior to oxidizing gas impingement, and the impinging oxidizing gas molecules cannot be adsorbed because electrons cannot be supplied from the sensor. Therefore, the conduction change is also small for diameters less than the critical thickness^7^.

**Figure 6:**
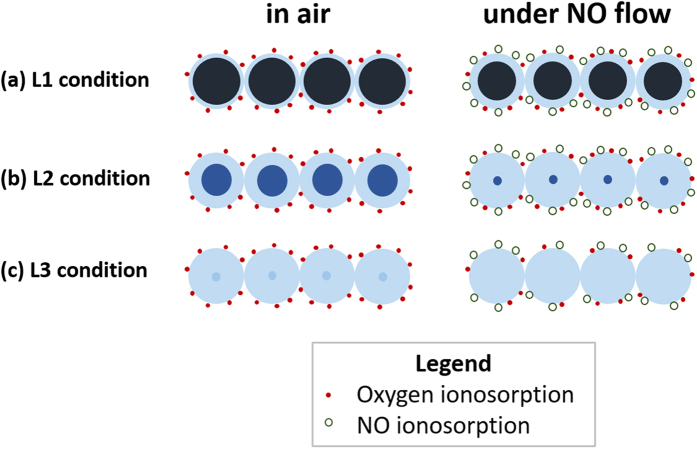
Schematic representation of the outer depletion region and inner conduction region in the connected particles of the sensor. **(a**–**c)** reflect the L1, L2, and L3 (and L4) conditions, respectively, of the different light intensity conditions in air and under a NO flow. The solid circles represent oxygen, and the open circles represent NO molecules adsorbed.

However, the size effect described above (i.e., the dependence of the response signal level on the nanowire diameter) is not an exact description. More precisely, the critical diameter for observation of the maximum response signal is a function of the depletion depth of the material, which varies with the free carrier concentration, temperature, and analyte gas concentration. For example, the critical diameter decreases as the electron concentration increases with higher UV irradiation intensity. Figure 6a schematically illustrates this L1 condition (case(a)) where a high UV light intensity produces a high EHP concentration and the depletion depth caused by oxygen adsorption in air is narrowed. When NO gas is introduced, the depletion depth will increase (transition from Fig. 3b to 3d). However, the conductance ratio before and after exposure to NO is not large owing to the extended neutral regions inside. Therefore, the sensor response level appears relatively small under the high UV intensity (L1).

Meanwhile, at a lower light intensity condition (L2, case(b)), the EHP population is less than that at the L1 condition, and the depletion depth is greater in air, as shown in Fig. 6b. The depletion depth increases further with NO flow, and the particles are under the nearly complete depletion condition, leading to a high resistance. Therefore, the conductance ratio is much greater than that from the high UV intensity (case (a)), comprising a higher response level. In the L1 and L2 conditions, the electron concentration generated in the oxide is sufficiently high to be supplied to all of the adsorbing O_2_ and NO molecules in the steady state. However, in the L3 (and L4) condition with a low EHP concentration, the particles are already nearly depleted in air. Therefore, when NO gas is introduced, the electron concentration is not sufficient to be supplied to all of the adsorbing NO molecules, and only a portion of the impinging NO molecules can be adsorbed, as schematically illustrated in Fig. 6c. As a result, a portion of the oxygen adsorption can be replaced by NO adsorption in the steady state. The model for the increasing response with light irradiation proposed by Mishra *et al*.^37^, which explained only the transition from case (c) to case (b) in Fig. 6 while the transition from case (b) to case (a) was missing.

### Transient adsorption/desorption kinetics of NO

The response and recovery curves in Fig. 5b were converted to Fig. 5c,d, respectively, for the detailed analysis of the adsorption-desorption kinetics of NO molecules on the In_2_O_3_ surface at RT under UV light in an air environment. The conversion of the plots was based on the conduction model structures that we proposed for nanowire sensor structures^7^,^8^. Briefly, the time-dependent conductance of the well-dispersed nanowire sensor is given by


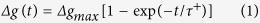


and


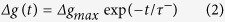


in the response and recovery cycles, respectively. These equations apply when a molecular adsorption/desorption process defines a sensing process. In other words, the sensor conductance change is only determined by the modulation of the surface depletion of the nano-sensor based on the ionosorption model^7^,^8^. In such case, the plots of Fig. 5c,d should reveal straight curves, and then, a clear characteristic set of response and recovery times (i.e., *τ*^+^ and *τ*^*−*^, respectively) can be obtained. However, the varying slopes were observed as a function of the progress of the response/recovery cycles in Fig. 5c,d, representing changes of *τ*^+^ and *τ*^*−*^ during the response/recovery cycles (or adsorption/desorption processes). Of particular note is that slopes varied more severely in Fig. 5c. This observation can be summarized as follows: i) UV light irradiation proportionally enhanced the response and recovery kinetics with the UV light intensity (i.e., progressively shorter time constants). Under a given light intensity, ii) the NO adsorption rate increased during the response cycle, but iii) the NO^−^ desorption rates were nearly constant (only slightly decreasing) during the recovery cycle. In the L1 condition, the response time constant (*τ*^+^) was initially approximately 100 s but decreased to approximately 10 s later in the final steady state. On the other hand, in the L4 condition with lower light intensity, *τ*^+^ changed from 8 min to 1.4 min. The recovery time constant (*τ*^*−*^) estimated in the L1 condition was approximately 4 min but approximately 22 min for the L4 condition.

With switching from the response cycle to the recovery cycle, the NO gas flow is suddenly shut down but O_2_ is continuously impinging on the surface during the response and recovery cycles. The linear curves shown in Fig. 5d indicate that the desorption kinetics were more or less controlled by NO^−^ desorption throughout the recovery cycles. The slightly higher recovery rate at the beginning may be originated from an initial rush of NO^−^ desorption when the NO flux stops. The transition is more dramatic in the response cycles. The slower initial transient in the response cycles shown in Fig. 5c represented hindered adsorption of NO combined with delayed desorption of O_2_^−^. When the population of the NO^−^ occupation becomes high in the later stage of the response cycle, the population of the O_2_^−^ occupation decreases, and the overall response kinetics are determined by the steady state adsorption/desorption rates of NO/NO^−^ and O_2_/O_2_^−^ (this is the condition in Fig. 3d). Because the adsorption reactivity of NO is much greater than that of O_2_, the initial slow NO adsorption rate shown in Fig. 5c was caused by the low partial pressure of NO. The relatively slower adsorption/desorption rates of NO/O_2_^−^ at a lower UV light intensity will increase at a higher UV light intensity, leading to a decreasing transition region and a reduced response time. Therefore, the shorter initial transient of the response at a higher UV light intensity was caused by the accelerated desorption of O_2_^−^ and accelerated NO adsorption. Therefore, it is elucidated that the non-constant response times during the cycles were derived from the competing kinetics between the adsorption/desorption of NO and that of O_2_.

### Sensor performance

**Figure 7:**
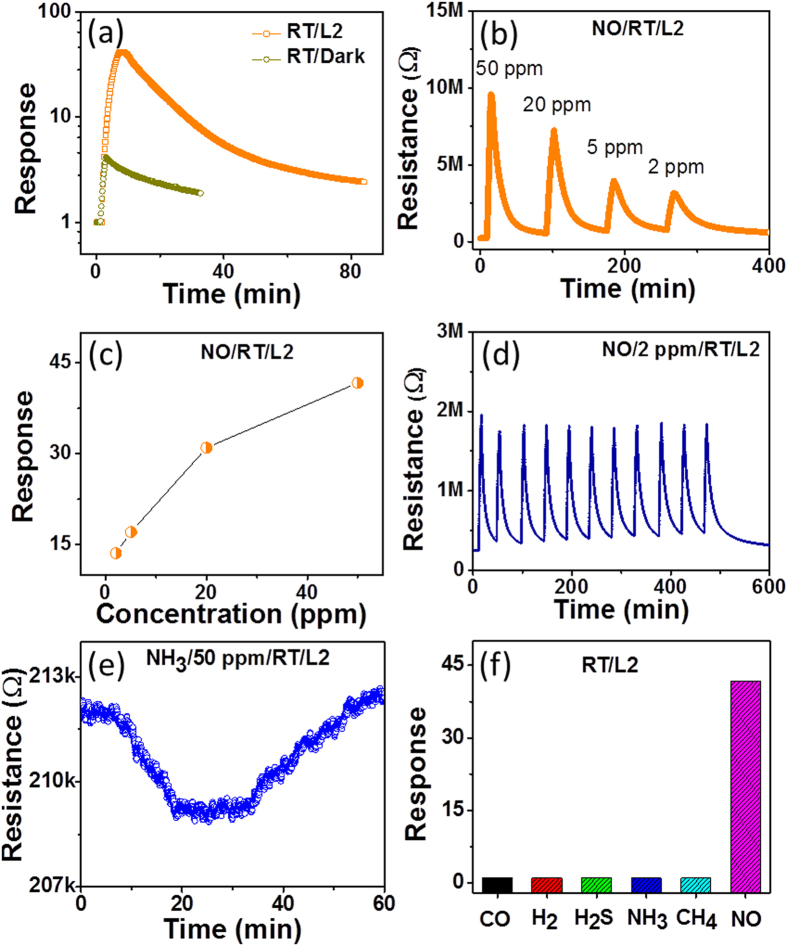
**(a)** Comparison of the gas sensing curves measured in the dark and in L2 at room temperature for 50 ppm NO. **(b,c)** Response to various NO concentrations measured under the L2 condition. **(d)** Repeatability of the sensor for 2 ppm NO under the L2 condition. **(e)** Gas sensing property of the In_2_O_3_ sensor for 50 ppm NH_3_ under the L2 condition at room temperature. **(f)** Comparison of the gas responses measured for 50 ppm NO, CO, H_2_, H_2_S, NH_3_, and CH_4_.

The sensing properties of 50 ppm NO gas in the L2 condition and in the dark are compared in Fig. 7a. A response of 41.7 under L2 conditions can be compared to a response of 4.3 in the dark. The sensing behavior that was measured at different NO gas concentrations (2–50 ppm) with the L2 condition is summarized in Fig. 7b,c, and the results indicate reasonably linear sensing signal changes as a function of the concentration. The sensing repeatability and long-term stability of the sensor are important parameters. Repeated measurements of the response-and-recovery to 2 ppm NO under the L2 condition are shown in Fig. 7d, and the results indicate excellent repeatability for 11 cycles. The gas selectivity of the sensor to 50 ppm of CO, H_2_, H_2_S, NH_3_, and CH_4_ gases at room temperature was also investigated under the L2 condition. As an example, Fig. 7e shows the typical response characteristics of the sensor to NH_3_ gas at RT under the L2 condition. The signal level was very small and unstable. Similar curves were obtained from the other reducing gases (i.e., CO, H_2_, H_2_S, and CH_4_), as summarized in Fig. 7f. The response results for the reducing gases can be compared to the response to 50 ppm NO (*S* = 41.7) to demonstrate the excellent selectivity to oxidizing NO gas under UV light irradiation at room temperature.

In the dark, even such a small response level could not be detected. Therefore, finite light effects assisted the combustion reactions of the reducing gases on the surface. The UV irradiation effect on the enhancement of the chemical reactions on the surface (as shown above in the sensing of the reducing gases) is much smaller than the temperature effect. The comparison indicates that UV irradiation is effective for the adsorption/desorption reactions of gas molecules but not for the chemical reactions of water molecule formation. The difference between the thermal energy and the photonic energy in the gas sensing should be further explored.

## Conclusions

An In_2_O_3_ nanostructure gas sensor was fabricated using the co-arc-discharge method to investigate the UV light irradiation effect on the adsorption/desorption of NO gas molecules at room temperature. The fabrication resulted in the morphology of a nanoporous thin film consisting of agglomerated In_2_O_3_ nanoparticles of various sizes. The In_2_O_3_ sensor exhibited a finite and stable response to NO gas at room temperature but the response and recovery were slow, as observed for other oxides. Therefore, UV light (365 nm, 3.2 mW/cm^2^) were irradiated to activate sensor materials by photon energy rather than using thermal energy. The sensing signal level as well as the response and recovery kinetics of NO sensing improved substantially. The fastest response time (i.e., 10 s) and recovery time (i.e., 4 min) were observed. The enhanced response kinetics under irradiation resulted from the enhanced desorption and adsorption of O_2_^−^ and NO, respectively, while the fast recovery was enabled by the enhanced desorption and adsorption of NO^−^ and O_2_, respectively. In addition, the highest response was observed at an intermediate UV light intensity, which confirmed the depletion model that explains the resistive sensing properties of nanostructures as proposed in the WO_3_ nanowire sensor. Furthermore, the effect of UV light irradiation on the combustion reactions was small as observed with sensing of reducing gas molecules. Therefore, the difference between the thermal and photon energies in relationship to the adsorption/desorption and chemical reactions could be clearly understood. The sensor also exhibited good repeatability for NO gas detection and good selectivity with respect to reducing gases. We believe our work systematically investigates the sensing behaviors of nano-sized oxide under the UV light irradiation and unveils the mechanism that comprises sensing properties, which will provide the guide to the development of the high-performance sensor.

## Methods

### Synthesis of In_2_O_3_ nanoparticles and characterization

Alumina substrates that were 2.5 mm × 2.5 mm × 0.23 mm were patterned with bar-type gold electrodes and used to fabricate the In_2_O_3_ nanostructures as sensor devices (Fig. S1b). The electrode-patterned substrates were ultrasonically and sequentially cleaned with acetone, methanol, and deionized water for 15 min each followed by blow-drying with nitrogen gas. The substrates were mounted on the inside wall of the arc-discharge chamber, as schematically shown in Fig. S1a. The active sensor area for In_2_O_3_ deposition, as shown in Fig. S1b, was defined by scotch-tape masking of the unwanted deposition area.

‘Co-arc-discharge’ is a process that we developed for the synthesis of highly crystalline metal and/or metal-carbon nanotube composites using a high temperature in the arc-discharging process. We previously used the method to fabricate carbon nanotube-metal oxide composites^39^. A hollow pure graphite tube with a length of 160 mm, outer diameter of 6.4 mm, and inner diameter of 3 mm was used as the carbon source and the vessel of the metal source, as schematically shown in Fig. S1c. The hollow tube was filled with indium powder (99.99% purity, Alfa Aesar). Arc-discharging of the graphite tube filled with the indium feedstock was performed for 10 min at an arc-discharge current density of 40 A cm^−2^ in a hydrogen atmosphere with a partial pressure of 5.3 × 10^3^ Pa. The substrates deposited with indium (but with carbon trace) were heat treated at 500 °C in air for 2 h to burn out the undesirable carbon nanoparticles and convert the indium to In_2_O_3_, which resulted in the fabrication of an In_2_O_3_ nanostructure sensor device, as schematically shown in Fig. 7b. The surface morphology and structure of the formed In_2_O_3_ nanostructure were investigated using field-emission scanning electron microscopy (FE-SEM; JSM 700F; JEOL) and X-ray diffraction (XRD, X’pert PRO-MPD, PANalytical, Netherlands).

### Sensor property measurements

The In_2_O_3_ nanostructure sensor device was mounted in a test chamber in which the temperature and gas flow can be controlled. A schematic diagram of the measurement setup is shown in Fig. S2. The resistance and gas sensing properties of the sensor were measured using a picoammeter/voltage source (Keithley 6487). A quartz window was equipped on top of the chamber for the illumination of light into the chamber from outside of the chamber. A UV light lamp that emitted at wavelength of 365 nm and a power of 4 Watts (Spectroline Model ENF-240C/FE, Spectronics Corporation, Westbury, New York, U.S.A.) was installed above the quartz window to irradiate the surface of the sensor. The intensity of the illumination (*I*) was estimated by the following equation: 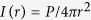, which indicates that the light intensity from a point source is inversely proportional to the square of the distance. *P* is the total power radiated from the light source, and *r* is the distance between the sensor and the UV light source. Therefore, we can control the UV light intensity illuminated on the surface of the sensor. Four different UV light intensities were tested as follows: L1 = 12.7 mW/cm^2^ with a 5 cm distance from the light source, L2 = 3.2 mW/cm^2^ at 10 cm, L3 = 0.8 mW/cm^2^ at 20 cm, and L4 = 0.35 mW/cm^2^ at 30 cm. We used the same sensor device to study the effect of UV light irradiation with different intensities.

In this experiment, the NO gas sensing properties were examined at a fixed concentration of 50 ppm unless otherwise specified. Different concentrations were only employed to test the working temperature effect and the reproducibility of sensing. Several reducing gases (i.e., CO, H_2_, H_2_S, NH_3_ and CH_4_) at a concentration of 50 ppm were only used to test the gas selectivity. The gas concentrations were determined by C(ppm) = C_std_(ppm) × f/(f + F), where f and F are the flow rates of the analyte gas and the carrier gas, respectively, and C_std_(ppm) is the concentration of the analyte gas in the gas cylinder. C_std_(ppm) was 1000 ppm balanced with nitrogen for all of the gases. Dry air was used as the carrier gas, and the gas flow rate was controlled by mass flow controllers. The response of the sensor (S) was defined by R_g_/R_o_ for the oxidizing gas (NO) and R_o_/R_g_ for the reducing gases (CO, H_2_, H_2_S, NH_3_, and CH_4_), where R_o_ is the standby resistance in a dry air environment prior to exposure to the analyte gases and R_g_ is the resistance upon exposure to the analyte gases at a specific concentration.

## Additional Information

**How to cite this article**: Chinh, N. D. *et al*. NO gas sensing kinetics at room temperature under UV light irradiation of In_2_O_3_ nanostructures. *Sci. Rep*. **6**, 35066; doi: 10.1038/srep35066 (2016).

## Supplementary Material

Supplementary Information
