# Supplementary material for: NO gas sensing kinetics at room temperature under UV light irradiation of In2O3 nanostructures
Source: Sci Rep. 2016 Oct 7;6:35066. doi: 10.1038/srep35066 (PMC5054382; doi:10.1038/srep35066)
Supplement: Supplementary Information [file srep35066-s1.pdf]

NO gas sensing kinetics at room temperature under UV light irradiation of In_2O_3 nanostructures

Nguyen Duc Chinh, Nguyen Duc Quang, Hyundong Lee, Truong Thi Hien, Nguyen Minh Hieu, Dahye Kim, Chunjoong Kim, and Dojin Kim*

Supporting Information

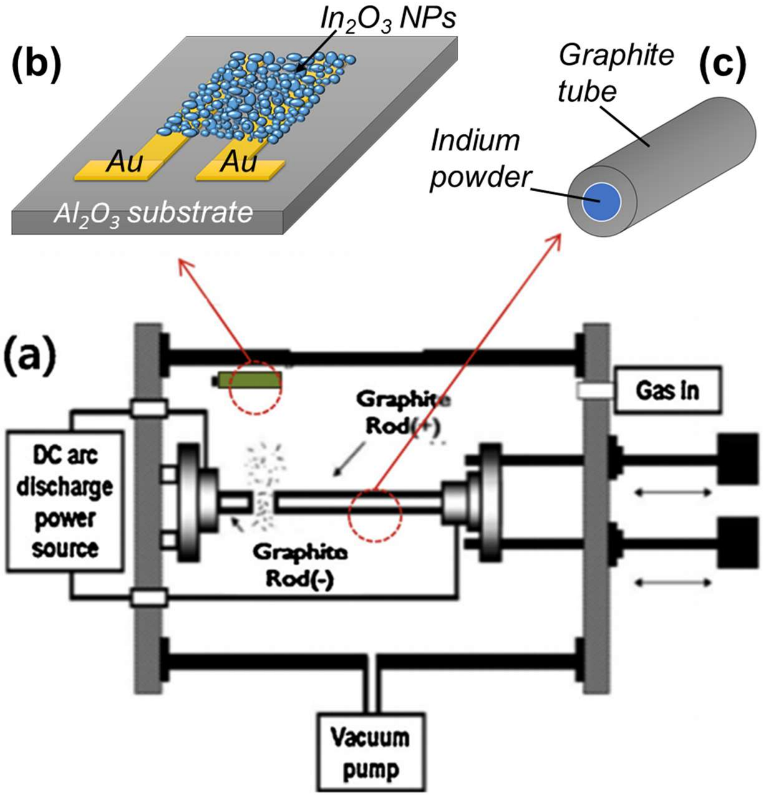

Fig. S1. (a) Schematic diagram of the arc-discharge system. The substrate is mounted on the inside wall of the chamber for the in situ synthesis of indium nanoparticles. (b) Schematic diagram of the sensor device deposited with In nanostructures on a Au electrode-patterned alumina substrate. (c) Graphite tube filled with In powder was used as the arc-discharging source.

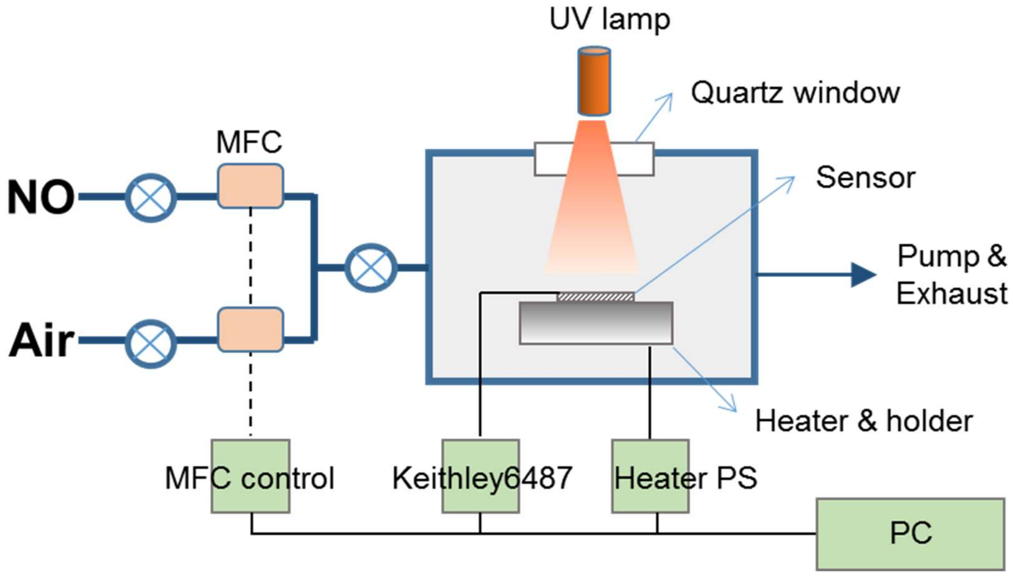

Fig. S2. Schematic diagram of the sensor measurement system.
